# Supplementary material for: Carrier-envelope phase on-chip scanner and control of laser beams
Source: Nat Commun. 2023 Aug 21;14:5068. doi: 10.1038/s41467-023-40802-z (PMC10442376; doi:10.1038/s41467-023-40802-z)
Supplement: Supplementary file 1 — Supplementary information [file 41467_2023_40802_MOESM1_ESM.pdf]

# Supplementary Information: Carrier-Envelope Phase On-Chip Scanner and Control of Laser Beams

**Václav Hanus[1*], Beatrix Fehér[1], Viktória Csajbók[1], Péter Sándor[1], Zsuzsanna Pápa[1,2], Judit Budai[2], Zilong Wang[3,4], Pallabi Paul[5,6], Adriana Szeghalmi[5,6] and Péter Dombi[1,2]**

1. *Wigner Research Centre for Physics, 1121 Budapest, Hungary*
2. *ELI-ALPS Research Institute, 6728 Szeged, Hungary*
3. *Physics Department, Ludwig-Maximilians-Universität, 85748 Munich, Germany*
4. *Max Planck Institute of Quantum Optics, 85748 Garching, Germany*
5. *Institute of Applied Physics, Abbe Center of Photonics, 07745 Jena, Germany*
6. *Fraunhofer Institute for Applied Optics and Precision Engineering, 07745 Jena, Germany*

hanus.vaclav@wigner.hu

## 1. Development of Ir/$Al_2O_3$ heterostructures and their properties

Ir/$Al_2O_3$ heterostructure (nanolaminate) coatings are grown using the atomic layer deposition (ALD) technique. Amorphous fused silica (FS) with an ultra-flat surface was used as substrates. The typical AFM (1 × 1 µm²) root-mean-square (rms) surface roughness is about 0.26 nm. Their cleaning was performed with a multi-stage, ultrasonic-assisted bath cleaning system (Elma Schmidbauer, Singen, Germany) with alternating surfactants and water ($H_2O$) baths, concluded by a deionized, ultra-pure $H_2O$ bath. The depositions were performed with a commercial SunALE R-200 Advanced ALD system (Picosun Oy, Masala, Finland) using iridium(III) acetylacetonate ($Ir(acac)_3$) and molecular oxygen ($O_2$), and trimethyl alumium (TMA) and water ($H_2O$) as precursors for Ir and $Al_2O_3$, respectively. A heatable wafer chuck ensures a substrate temperature of 380 °C. One Ir ALD cycle consists of 6 s of $Ir(acac)_3$ pulse, 60 s of purge, 2 s of $O_2$ pulse, and 6 s of purge with molecular nitrogen ($N_2$) as purging gas. The corresponding ALD process parameters for $Al_2O_3$ are 0.1 s TMA pulse, 4 s $N_2$ purge, 0.2 s $H_2O$ pulse and 4 s $N_2$ purge. The growth per cycle (GPC) of Ir and $Al_2O_3$ are 0.6 Å/cycle and 0.9 Å/cycle respectively. The development and properties of ALD-grown Ir coatings and Ir/$Al_2O_3$ nanolaminates are reported in detail in previous articles [1–3]. The Ir/$Al_2O_3$ ratio is precisely controlled by the number of ALD cycles, which allows to tailor the optical and electrical properties of the heterostructures. Here, a composition with 32 cycles of Ir separated by 35 cycles of $Al_2O_3$ and a total thickness of 226.5 nm is used.

The presence of iridium layers is beneficial in terms of the maximum attainable signal $J_0$ of strong-field CEP-dependent ultrafast currents. In FigSI. 1 we show how the signal gain increases from bare $Al_2O_3$ ALD to iridium containing ALD coating when exposed to similar field strengths. Moreover, the selected nanolaminate medium shows a very good trade-off between stability and magnitude of the signal, see below. We surmise that these advantageous properties of this material stem either from its large nonlinear susceptibility $\chi^{(3)}$ [1] or from a conduction band that is not empty. The former increases coupling between the valence and the conduction bands, thus, interband contribution to the current [4,5], while the latter enhances the contribution of intraband oscillations to the current [6,7]. Additionally, the conductivity of the several-atom-thin iridium layers might contribute to the fast recovery between the laser pulses.

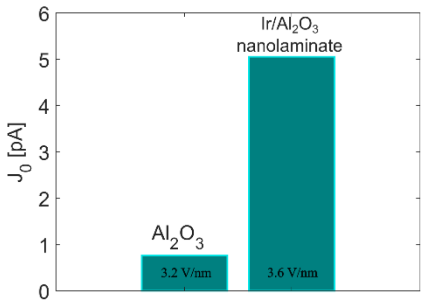


FigSI. 1 Effect of iridium heterostructure (nanolaminate) coating on the maximum attainable signal $J_0$ of strong-field CEP-dependent ultrafast currents.

## 2. Equivalence of measured lock-in phase and CEP

Our CEP detection principle of beams phase-locked to non-zero $f_{CEO}$ relies on the equivalence between the spatial dependence of the detected lock-in phase and CEP. For better understanding of validity of this equivalence we put an illustration in FigSI. 2(a). Two pulse trains on the picture can also represent two different spatial points with respective CEP offset of 45°. As soon as the CEP is different between these two points in space also the time-of-arrival of two same waveforms will differ by $f_{CEO}^{-1}\times(45/360)$ and the electrical signal oscillating at $f_{CEO}$ (green line) will be offset by the same amount. This will project into the measured lock-in phase $\varphi_J$. Thus. change of $\varphi_J$ is a direct measure of spatial change of CEP.

Next, the validity of CEP change detection scheme can be demonstrated performing a wedge scan (slow adding of glass in the beam line) that introduces continuous rotation of CEP, i.e. CEP offset in the beamline as replicating method in [8]. In FigSI. 2(b) we show a result of such a scan and one can see about four oscillations of the in-phase $J_{\cos}$ current component obtained with lock-in amplifier on the distance that corresponds to 8π shift, i.e., 4 CEP cycles. The reason for seeing slightly more than four CEP oscillations on the 8π interval we attribute to the approximate nature of the glass thickness to CEP conversion for a broadband laser pulse. The density of oscillation can be directly expressed in terms of phase, i.e. $\varphi_J = \arctan(J_{\sin}/J_{\cos})$, which is plotted in FigSI. 2(c), and it is well linear on the whole range of the wedge (glass) scan.

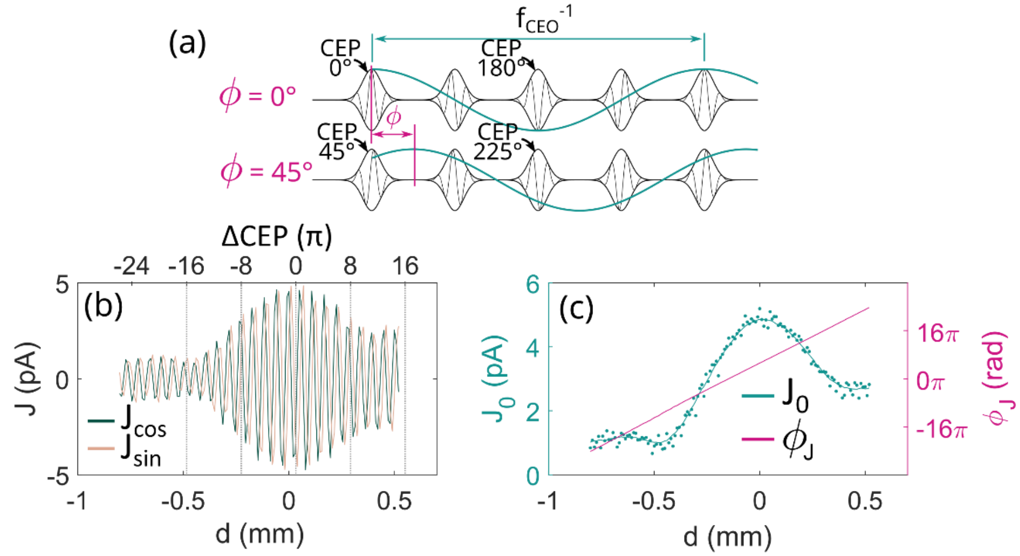


FigSI. 2 (a) Illustration of measured lock-in phase $\varphi_J$ and CEP equivalence. The two depicted pulse trains differ in CEP offset by 45°. This projects to the offset of the current oscillation (green line) in the connected circuit leading to the change in the detected lock-in phase $\varphi_J$ by 45°. (b) CEP change sensitivity of a current generated in Ir:$Al_2O_3$ target. The plot shows the oscillation of the measured in-phase $J_{cos}$ and quadrature $J_{\sin}$ components that acquired with lock-in amplifier as amount of glass *d* is added into a beamline. Secondary abscissa shows an estimated change of CEP calculated from *d*. (c) Change of magnitude of the current $J_0$ (green) and its phase $\varphi_J$ (pink) that corresponds to the offset of the phase in the pulse train as indicated in (a).

## 3. Stability and robustness of CEP measurement

We have investigated the stability of the CEP measurement by investigating the probe design from Fig. 1 in the main manuscript. To show that the chosen nanolaminate of $Al_2O_3$ and Ir performs well we made the same electrodes on ALD-deposited $Al_2O_3$ without iridium content. We made same probe on this substrate and tested it under same conditions as the probe deposited on the heterostructure. In

FigSI. 3 we show the signals from investigated probes. We can see that only in case of $Al_2O_3$:Ir probe the stability of the measurement is good enough to make a reliable CEP measurement. During 300 s long measurement with 3 Hz acquisition rate the magnitude of current $J_0$ is constantly settled on a value of 218±17 fA. The phase is accompanied by little oscillation lower than 10° peak-to-peak. However, in one minute interval (180 samples) the standard deviation of the signal is only 3.5°. The noise sample from the $Al_2O_3$:Ir probe was measured when the laser illuminated the probe but the lock-in reference loop was disengaged. The current noise was on the order of 20 fA, i.e. 10 % of the

typical signal. As expected, the phase jumps quasirandomly. The $Al_2O_3$ reference sample without iridium content does not perform very well. Although magnitude of current reaches much higher it is not stable. Also, the noise with the reference loop disengaged is much higher.

The measured phase $\varphi_J$ has a weak dependence on the electric field the probe is exposed. This is well visible while performing a power scan (beam attenuation with a reflective ND filter) on a stationary CEP-probe that shows some linear slope of the phase as a function of the electric field, see FigSI. 4(a). This can have some consequences on the CEP scans. As the strength of laser field in the vicinity of beam focus changes fast, there can be some crosstalk between the current and phase. Fortunately, the measurements show that the crosstalk is not higher than -9°nm/V as obtained from a linear fit of the data in FigSI. 4(a). In order to compensate for this crosstalk, we constructed a correction based on the measurement that we used in the data post-processing. The correction is represented by a function mapping the measured current $J_0$ on a phase, see FigSI. 4(b). Measured maps $J_0(x,y,z)$ then allow us to make maps of relative offset that we apply on the measured maps of $\varphi_J(x,y,z)$.

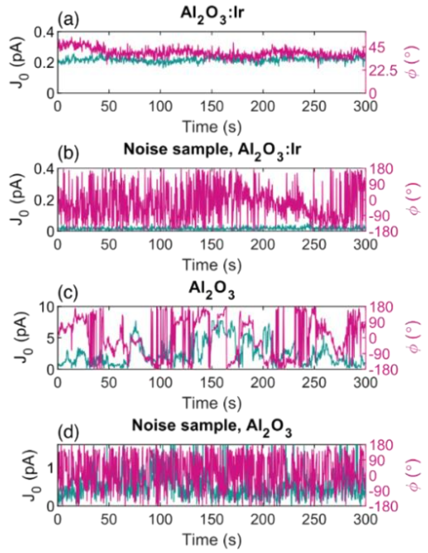


FigSI. 3 t = 300 s measurement with 3 Hz acquisition rate of current $J_0$ and phase $\varphi$ for the $Ir/Al_2O_3$ nanolaminate and reference ALD-deposited $Al_2O_3$. (a, c) and (b, d) show measurements with the lock-in reference loop active and disengaged respectively.

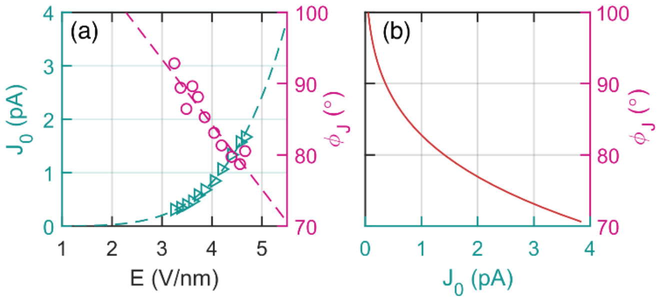


FigSI. 4 (a) Measured lock-in current magnitude $J_0$ (green triangles) and phase $\varphi_J$ (pink circles) as they are generated during the illumination of the CEP-probe. The CEP-probe and beam is kept stationary, while the power of the beam is changed. Dashed line represent power and line fits respectively. (b) Line shows relation between the fits of measured current and phase $\varphi_J(J_0)$ which is used as a correction to the measured CEP scans.

## 4. Focal spot measurement

The tight focusing geometry of expanded beam is prone to a variety of aberrations. To ensure that the beam is as close as possible to Gaussian $TEM_{00}$ mode we have characterized it around the focus with an in-situ knife edge method. In this method the beam is cut using a horizontal and vertical edge of the electrode, see Fig. 1(b) of main manuscript, in

a range of positions along the optical axes $z$. The derivative of the integrated laser power throughput as a function of the edge insertion is fitted with a Gaussian to get its waist size $w(z)$. Subsequently, obtained waists $w$ are fitted with the waist function

$$w(z) = w_0 \sqrt{1 + \frac{\lambda^2 (z - f_0)^2}{\pi^2 w_0^4}} \quad (1)$$

to get the waist of the beam $w_0$ and the focal position $f_0$. $\lambda$ = 800 nm is the central wavelength. In FigSI. 5 we show the results of the knife edge measurements of beams characterized in terms of CEP in Figs. 2 and 4 of the main manuscript.

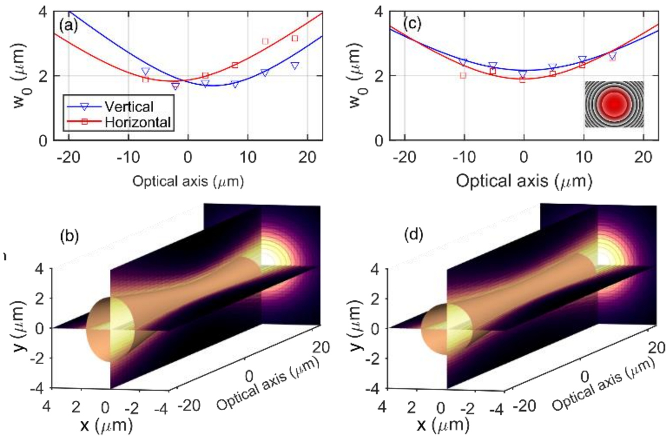


FigSI. 5 (a) Results of knife edge characterization of the laser beam along the focus for the beam presented in Fig. 3 of main manuscript. Plot shows vertical (triangles) and horizontal (squares) beam sizes resulting from Gaussian fits of the knife edge measurement data as a function of distance along the optical axis. Points are fitted with a waist function assuming a Gaussian beam. Beam waists $w_0$ obtained from the fits are 1.7 and 1.8 µm and focal positions $f_0$ are 4.2 and -2.6 µm for vertical and horizontal focus respectively. (b) Reconstruction of the beam intensity profile (assuming Gaussian beam, normalized to on-axis intensity) as measured with the knife edge method in (a). The orange isosurface is drawn at the value of $e^{-1/2}$ of the on-axis intensity. (c, d) shows same as (a, b), but for measurement in Fig. 5 of main manuscript. Beam waists $w_0$ obtained from the fits are 2.2 and 1.9 µm and focal positions $f_0$ are 0.3 and -0.2 µm for vertical and horizontal focus respectively. Inset depicts applied SLM pattern.

## 5. Evaluation of model of focal CEP distribution

In FigSI. 6 we show a few selected examples of CEP distribution in the vicinity of the focus as evaluated with the formula Eq. 12 in [9]. We would like to highlight the property of chirped beam that forms plateaus in the post- and

pre-focal regions for negative and positive values of $g$ respectively. Another distinct feature is the crest along the optical axis that is pronounced for the negative chirp.

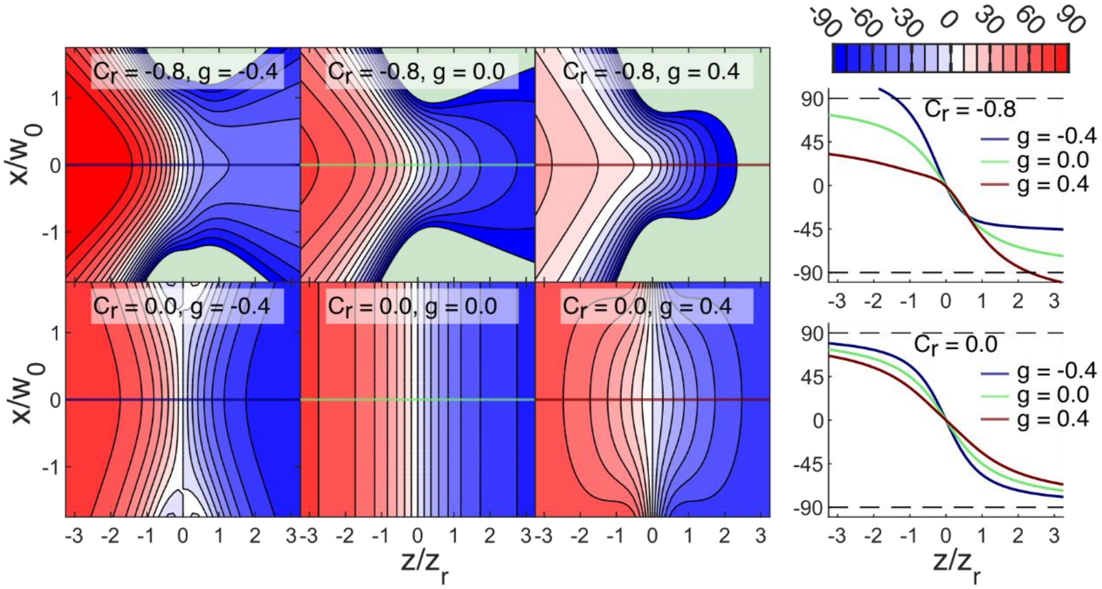


FigSI. 6 Selected examples of calculated CEP landscape in the vicinity of focus according to eq. (2) in main manuscript (Methods). Waist and Rayleigh length of the considered beam is $w_0$ = 1.5 μm and $z_r$ = 8.8 μm respectively. $\gamma$ is set to zero in all depicted cases. 1D lines represent lineouts along the optical axis $z$.

## 6. Spectral characteristic of laser pulse

Related to the studies of change of CEP landscape as a function of laser pulse chirp, we investigated the spectral phase of the laser pulses. We performed a measurement of the laser spectrum and a d-scan trace to obtain the spectral phase. Once the d-scan trace is reconstructed we obtain the spectral phase denoted as $d$ = 0.0 mm, see the spectrum (yellow) and the phase (black) in FigSI. 7. We fitted the phase and obtained the polynomial expansion centered at the laser central frequency 2.35 rad/fs (800 nm). The coefficient of the fit at the second power $p_2$ gave us the relative chirp values as $C_r = 4\ln(2)p_2/\tau^2$. Interestingly, despite the pulse in state $d$ = 0.0 mm giving the highest currents $J_0$ from the probe there is still a residual phase curvature which in turn leads to a non-zero value of $C_r$ = -0.8 at the point of the central frequency. This justifies the use of negative chirp in the model for the measurements performed with pulse that yielded the highest currents $J_0$. We recall that the model from [1] used for fitting the CEP landscape measurements does not consider the whole spectral phase but deals only with a point estimate at the laser central frequency. This is how we understand slight mismatch between the $C_r$ values reached from the d-scan reconstruction and CEP landscape fitting.

Additionally, we show how the point estimate of chirp changes by adding fused silica in the beamline. We calculated an addition to the reconstructed phase of the equivalent of thickness $d$ of the glass, resulting phase is showed in FigSI. 7. One can see that the phase curvature, hence the evaluated $C_r$ can be adjusted and even increased by adding 0.5 mm of fused silica as the $C_r$ in this case reduces almost to zero.

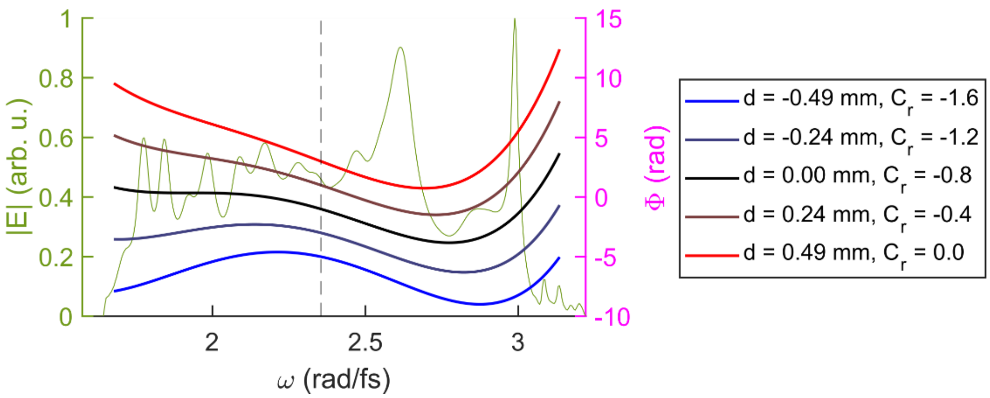


FigSI. 7 Spectral characteristic of the laser used to test the CEP-probe. Measurement of laser spectral intensity |E| (yellow line) is showed with the reconstructed spectral phase (black line) as obtained from the d-scan method. The phase profiles represented by color range from blue to red are calculated assuming the pulse has acquired additional phase due to propagation in fused silica of thickness *d*. $C_r$ is the relative chirp as obtained from polynomial fits of the phase. Vertical dashed line highlights the central frequency 2.35 rad/fs.

## 7. Chromatic aberration of SLM

Measurement of a beam size as a function of the color components of the spectra clearly shows the non-trivial chromatic characteristic of the few-cycle laser beam under investigation. Moreover, on account of the SLM, that is by definition causing the wavelength dependent phase shift, we are able to change the beam size vs. wavelength characteristic. In FigSI. 8 we show a measurement of beam sizes (FWHM in intensity) as a function of frequency (wavelength). The measurement was done with a set of 6 interference filters (10 nm FWHM of transmission bandwidth) at a distance of 1.370 m from the SLM. One can see that the dependence of beam size on frequency changed for beams produced with no SLM pattern and with SLM lensing pattern. The beam presented in FigSI. 8 was used to produce the measurements in the main manuscript in Figs. 2 and 4.

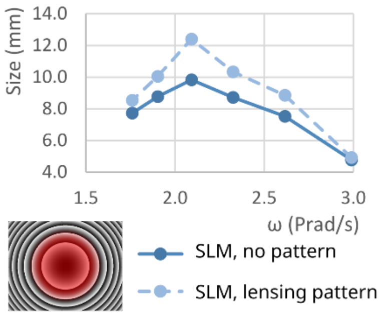


FigSI. 8 Beam sizes after the SLM as measured at distance of 1.370 m using a camera and narrow-band interference filters. The image shows the lensing pattern applied to the SLM to increase divergence of the beam. Real size of the image (i.e. SLM chip) is 16×12.8 mm. Red spot approximately illustrates the size of the used beam.

## 8. Discussing capabilities of SLM for CEP sculpting for few-cycle lasers based on hollow-core fiber postcompression

We analyzed the possibility to apply the SLM method for few-cycle lasers based on hollow-core fiber postcompression. The key is to address the chromatic aberrations being present in the fibre output beam as they also influence parameters $g$ and $\gamma$. Therefore, the SLM should be able to manipulate a certain range of color-dependent phase shifts across the size of the beam. As a representative example, we can consider laser beam characteristics similar to the case presented by Alonso et al. [10]. In FigSI. 9(b) we mimic the case from Fig. 5(b) of that article. The figure shows the wavefront across the beam radius for two selected colors: 600 and 900 nm. One can see that the blue components are more diverging, and the range of the wavefront curvature reaches about 20 radians with a difference of 7.5 radians between the two colors. Applying a curved pattern to the SLM would cause a retardation while in the center the blue components would be more retarded than the red ones, see FigSI. 9(a). (Note that in this example, the

pattern is an inverted version of the one used in Fig. 5 of the main manuscript). Consequently, the chromatic aberration can be reduced by application of a curved pattern on the SLM as the SLM induces enough phase shift to invert the difference between the color components, see panel FigSI. 9(c), where the phase difference (black line) between the two colors is flatter than in FigSI. 9(b). At the same time, the SLM pattern can be sculpted further in order to achieve a custom CEP spatial distribution in a similar manner which is presented in Fig. 6 of the main manuscript. In case a system possesses greater aberrations than considered in this example, one would need to find ways to precompensate this by other means, e.g., with lenses.

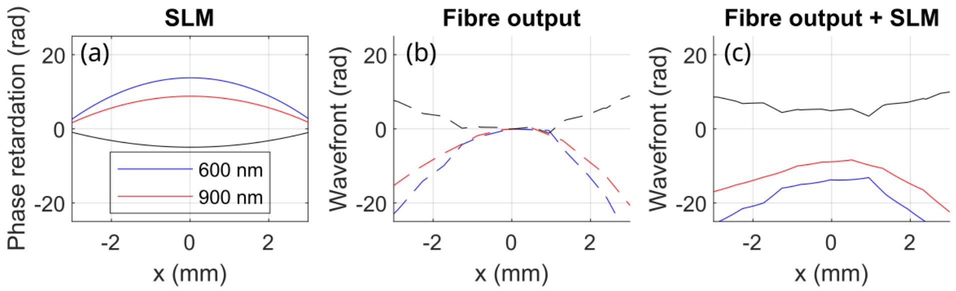


FigSI. 9 Proposal for compensating the chromatic aberration of the typical output of a hollow-core fibre pulse compressor. (a) Lensing pattern on the SLM with high retardation in the middle retarding the 600-nm component (blue line) more than the 900-nm one (red line), while at the outer part of the beam the retardations are equal. The black line shows the difference between these two components. (b) A representative wavefront of a hollow-core fibre compressor output, mimicking data from Alonso et al. [10] (c) Wavefront resulting from an addition of the SLM-induced retardation and fibre output. The phase difference at the outer parts of the beam were decreased by the action of the SLM.

## 9. Laser beam polarization state and probe's linear polarization-orientation dependence

One can expect a polarization sensitivity of the CEP-scanning probe. With this having in mind, we first carefully characterized a polarization state of our linearly polarized laser beam. We performed a rotation scan with a polarizer to obtain the extinction ratio between the minimum and maximum transmitted power yielding 0.2 %, see FigSI. 10(a). Thus, the investigated laser beam was having a clean linear polarization. Then, we investigated the polarization rotation response of the probe. We define $\theta$ as the angle between the polarization direction and the sample axis defined as a line connecting the tips of the two opposing electrodes. To measure the dependence of the CEP-dependent current $J_0$ as a function of $\theta$ we rotated step-wise the sample about its axis and acquired the current as a function of spatial coordinates $x$ and $y$, and found a maximum of obtained values $J_0(x,y)$. This is necessary as it is difficult to overlay the center of the sample with the rotation axis. As a result, we observe a decreasing current following approximately the relationship $J_0 \propto \cos(\theta)$, see FigSI. 10(b).

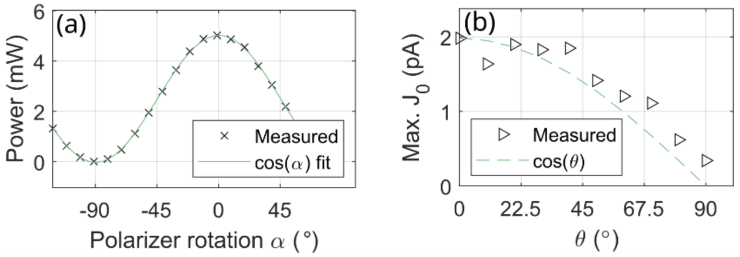


FigSI. 10 (a) Measurement of polarization state of the laser beam used in the experiment. The plot shows the measured power after a polarizer as a function of its rotation $\alpha$. $\alpha = 0$ is a horizontal direction in the lab frame. Points are fitted with a cosine function (green line). The extinction was determined to be 0.2 %. (b) Extinction of the CEP-dependent current $J_0$ as a function of the sample orientation $\theta$. The vector connecting the two opposing electrodes is parallel to the laser polarization direction when $\theta = 0°$. The measured points are accompanied with a cos($\theta$) function as an eye guide.

## 10. References


1. P. Schmitt, P. Paul, W. Li, Z. Wang, C. David, K. Hanemann, N. Felde, A. Munser, and M. F. Kling, "Linear and Nonlinear Optical Properties of Iridium Nanoparticles by Atomic Layer deposition," 1–14 (2023).

2. P. Schmitt, V. Beladiya, N. Felde, P. Paul, F. Otto, T. Fritz, A. Tünnermann, and A. V. Szeghalmi, "Influence of substrate materials on nucleation and properties of iridium thin films grown by ald," Coatings **11**, 1–23 (2021).

3. P. Paul, P. Schmitt, V. V. Sigurjónsdóttir, K. Hanemann, N. Felde, S. Schröder, F. Otto, M. Gruenewald, T. Fritz, V. Roddatis, A. Tünnermann, and A. Szeghalmi, "Atomically Thin Metal–Dielectric Heterostructures by Atomic Layer Deposition," ACS Appl. Mater. Interfaces (2023).

4. M. Hazra, P. Paul, D. Kim, C. David, S. Gräfe, U. Peschel, M. Kübel, A. Szeghalmi, and A. N. Pfeiffer, "Nonlinear polarization holography of nanoscale iridium films," arXiv **2211**, 03436 (2022).

5. P. Jürgens, B. Liewehr, B. Kruse, C. Peltz, D. Engel, A. Husakou, T. Witting, M. Ivanov, M. J. J. Vrakking, T. Fennel, and A. Mermillod-Blondin, "Origin of strong-field-induced low-order harmonic generation in amorphous quartz," Nat. Phys. **16**, 1035–1039 (2020).

6. P. Földi, M. G. Benedict, and V. S. Yakovlev, "The effect of dynamical Bloch oscillations on optical-field-induced current in a wide-gap dielectric," New J. Phys. **15**, (2013).

7. J. Reislöhner, D. Kim, I. Babushkin, and A. N. Pfeiffer, "Onset of Bloch oscillations in the almost-strong-field regime," Nat. Commun. **13**, 7716 (2022).

8. V. Hanus, V. Csajbók, Z. Pápa, J. Budai, Z. Márton, G. Z. Kiss, P. Sándor, P. Paul, A. Szeghalmi, Z. Wang, B. Bergues, M. F. Kling, G. Molnár, J. Volk, and P. Dombi, "Light-field-driven current control in solids with pJ-level laser pulses at 80 MHz repetition rate," Optica **8**, 570 (2021).

9. M. A. Porras, Z. L. Horváth, and B. Major, "Three-dimensional carrier-envelope-phase map of focused few-cycle pulsed Gaussian beams," Phys. Rev. A **98**, 063819 (2018).

10. B. Alonso, M. Miranda, F. Silva, V. Pervak, J. Rauschenberger, J. San Román, Í. J. Sola, and H. Crespo, "Characterization of sub-two-cycle pulses from a hollow-core fiber compressor in the spatiotemporal and spatiospectral domains," Appl. Phys. B Lasers Opt. **112**, 105–114 (2013).
